# Ureteral Strangulation by Fibrosis: A Cold Case Report of Ormand's Disease

**DOI:** 10.1155/2011/302963

**Published:** 2011-07-10

**Authors:** Amarpreet Sandhu, Leslea Brickner, Mark Chen

**Affiliations:** Department of Medicine, Kaiser Permanente, Oakland, CA, USA

## Abstract

Retroperitoneal fibrosis or Ormand's disease is rare in incidence and clinically elusive to diagnosis until obstructive uropathy clinically manifests by the mechanism of ureteral fibrotic strangulation and acute renal failure. We encountered a 50-year-old woman with months of nonspecific abdominal pain and presented with signs and symptoms of acute renal failure. Laboratory data was significant for blood urea nitrogen 47 mg/dL and creatinine of 8.47 mg/dL. Renal ultrasound revealed bilateral hydronephrosis and an abdominal computed tomogram confirmed an abnormal soft tissue retroperitoneal confluence that encased the pelvic vessels. Urologic consultation was requested and bilateral ureteral stents were placed with relief of her obstructive uropathy. Five days after ureteral stenting her creatinine dropped to 1.64 mg/dL. One month later patient underwent ureterolysis with biopsy showing fibroblast proliferation consistent with acute and chronic inflammation. By ruling out infections and malignancy, the final diagnosis was made to be idiopathic retroperitoneal fibrosis.

## 1. Introduction

Ormand's disease or retroperitoneal fibrosis encompasses a range of diseases characterized by fibro inflammatory tissue near the abdominal aorta and the iliac arteries that extend craniocaudally into the retroperitoneum. The first historical description of this clinical entity was reported in 1905 by a Cuban urologist, Joaquín Albarrán, but was not formally documented until 1948 by an American urologist, John Kelso Ormand [1].

Incidence is reported as 0.1 per 100,000 person years with a 3 : 2 male : female predominance. Nearly two-thirds of cases are classified as idiopathic. Secondary causes include but are not limited to medications, chemotherapy, and certain infections [1]. Symptoms are often nonspecific back, flank, or abdominal pain and constitutional symptoms of fatigue, weight loss, anorexia, and low-grade fevers. There are no standardized diagnostic criteria for retroperitoneal fibrosis. The diagnosis is often exclusive until obstructive pathology manifests.

Because little is known about the pathogenesis of idiopathic retroperitoneal fibrosis, treatment has not been well defined. Evidence confirms a local fibrotic process however systemic inflammation may also be at play, supported by the finding of elevated inflammatory serum markers in a majority of patients with this disease. However this case is classic in regards to affected presenting age range, clinical symptoms, and urographic features reigniting discussion and collaboration from the disciplines of rheumatology, urology, and general internal medicine.

## 2. Case Report

A 50-year-old woman with a past medical history of hypertension treated with lisinopril and chronic obstructive pulmonary disease was seen by her primary care physician five months prior to admission presenting with lower abdominal pain. Her review of systems was negative at the time except for decreased bowel movements. After being diagnosed with constipation the patient presented again one month later with lower abdominal pain localized to the left lower quadrant and low grade fevers. The patient was sent home with ciprofloxacin and metronidazole for 14 days for suspicion of diverticulitis.

**Figure 1:**
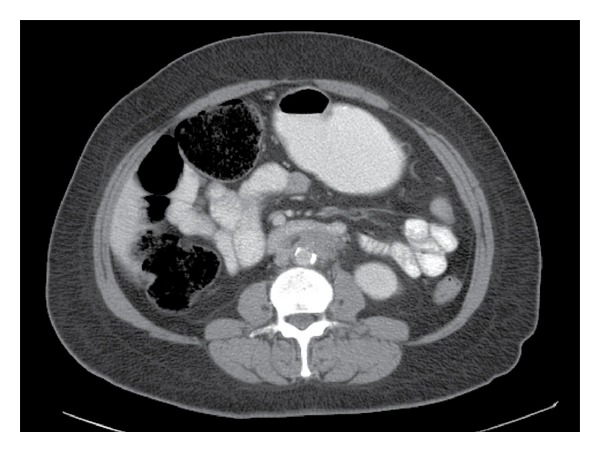
Abdominal computed tomography featuring the aorta with surrounding soft tissue mass.

Though the symptoms abated somewhat the patient again presented with pain suggestive of cholecystitis. She underwent an abdominal computed tomography scan (Figure 1) which incidentally identified a confluent soft tissue mass tracking along the retroperitoneum encasing the surrounding vessels. Seemingly not related to her current symptoms the patient was asked to follow up with another radiological scan in a few months. A hepatobiliary iminodiacetic acid scan confirmed cholecystitis and a cholecystectomy was performed. 

One month later though her initial pain had resolved after the surgery she complained of a constant low grade pain described as progressively worsening, nonradiating, and epigastric accompanied by nausea, vomiting, fatigue, and anorexia for 5 days. She reported recent decrease in urine output and 20 pound weight loss over the last 4 months. The patient denied fevers, chills, dysuria, diarrhea, constipation, or blood in stool or urine.

Physical Exam with blood pressure 162/91 mmHg, pulse 79 bpm, temperature 98°F (36.7°C), respiratory rate 18, and SpO2 99% on room air. She was alert, well appearing, and in no distress, oriented ×3. Abdomen was nondistented, bowel sounds were normal, slight epigastric tenderness without hepatosplenomegaly, rebound, or guarding. Rest of her exam including neck, chest, cardiac, back, and extremities exam was within normal limit. 

Her pertinent labs included Sodium 146 mEq/L, Potassium 5.1 mEq/L, Creatinine 8.18 mg/dL (baseline 0.8 mg/dL), white blood cell count 10.4 K/uL, hemoglobin 13.1 g/dL, hematocrit 40.5%, and platelets 383 K/uL (Table 1).

**Figure 2:**
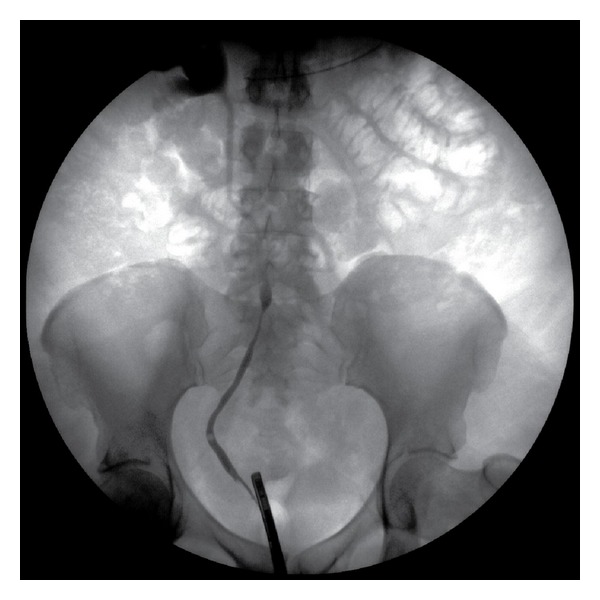
Retrograde pyelogram demonstrating evidence of extrinsic compression and medial deviation of the right ureter.

In the emergency department, she was able to spontaneously urinate but amount was subjectively decreased. The mild bilateral hydronephrosis on renal ultrasound suggested obstructive pathology. A urologic consultation was requested for suspicion of postobstructive uropathy. The next morning she underwent a retrograde pyelogram demonstrating bilateral hydronephrosis, extrinsic compression, and medial deviation of the ureters, particularly on the right (Figure 2). Ureteral stents were placed with relief of her obstructive uropathy.

Her renal function and electrolytes were followed for the risk of electrolyte abnormalities due to postobstructive diuresis. Over the course of days her renal function returned to baseline and she was discharged with a plan for surgical ureterolysis and open biopsy one month later. Surgical pathology confirmed tissue consistent with inflammation and ruled out any evidence of malignancy thus making the final diagnosis idiopathic retroperitoneal fibrosis (Figure 4). The patient remained asymptomatic for 1 year and then lost followup.

## 3. Discussion

Retroperitoneal fibrosis or Ormand's disease is rare in incidence and clinically elusive to diagnosis. Patient history most often reveals a chronic history of abdominal pain and alternative explanations for their symptoms. Diagnosis is most often delayed until obstructive uropathy clinically manifests by the mechanism of ureteral fibrotic strangulation and acute renal failure [1].

Our patient, however, did exhibit the classical triad of proximal hydroureteronephrosis, medial deviation, and extrinsic compression of the ureters characteristic of Ormand's disease.

Current theories suggest two possible mechanisms of chronic periaortitis to explain the localized inflammation found in the retroperitoneum (Figure 5).

- Ceroid mediated inflammation: ceroid is a lipoproteic polymer that results from low-density lipoprotein oxidation within plaque macrophages.
- Small vessel vasculitis in the aortic adventitia causing an inflammatory process weakening the aortic wall with medial thinning, atherosclerosis, and localized fibrosis [1].

The pathogenesis of idiopathic retroperitoneal fibrosis may also be multifactorial as the systemic markers of inflammation (ESR, CRP) are elevated in up to 70% of patients [2] (Table 2). A case control study found that the disease is related with HLADRB1*03 [1]. This allele is also linked to type I diabetes mellitus, myasthenia gravis, and systemic lupus erythematosus.

**Figure 3:**
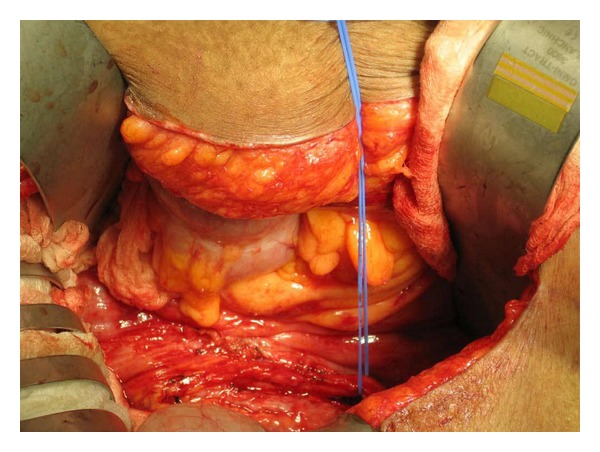
Intraoperative photograph of the right ureter isolated and freed from the surrounding fibrosis.

**Figure 4:**
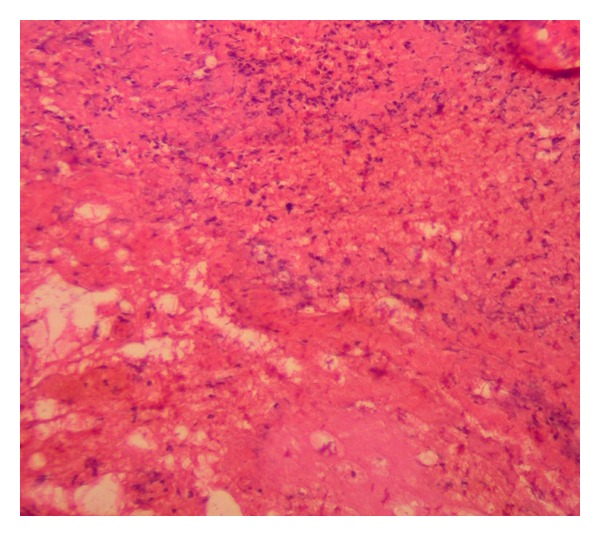
Surgical pathology showing inflammation.

**Figure 5:**
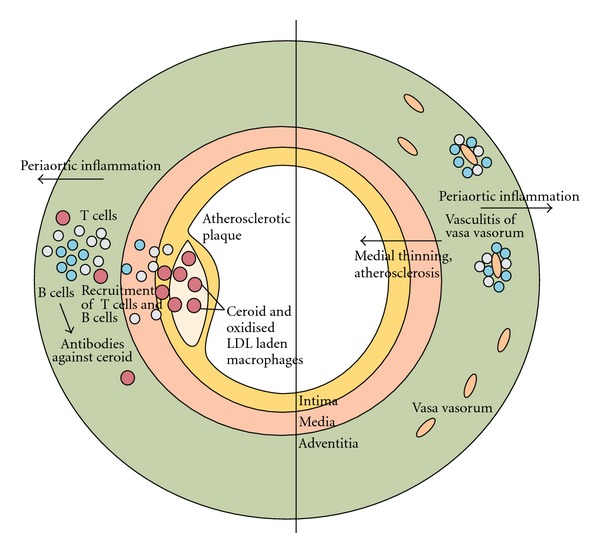
Two potential pathogenetic mechanisms of chronic periaortitis [1].

Surgical ureterolysis is optimal in freeing the encased ureters. The fibrotic tissue, often described by urologists as “woody box” in texture and appearance, can aid in pathological diagnosis (Figure 3) [3]. Medical therapy aims to cease the systemic inflammatory response, reduce retroperitoneal inflammation, and inhibit progression of fibrosis. Given the rare nature of this disease, definitive treatments have not been well established. Steroid therapy as well as immunosuppressive agents (cyclophosphamide, azathioprine) have shown promise in few studies.

Over a century has passed since Albarrán first described his findings of retroperitoneal fibrosis. Although many advances in medicine have evolved over this time period, Ormand's disease remains extremely difficult to diagnosis. See Table 3 for differential diagnosis of retroperitoneal fibrosis [4]. As in the case of this patient, physical examination and early presenting symptoms are usually inconclusive allowing alternative diagnoses to be more likely. Even when a diagnosis of retroperitoneal fibrosis is suspected a clear etiology is almost never found. However much has been learned regarding the inflammatory nature of its pathogenesis which in turn contributes to important lessons in our understanding of complex idiopathic diseases and the therapeutic options we can offer these patients.

## Figures and Tables

**Table 1:** Measurement of serum creatinine during hospitalization.

	Admission	Day 3	Day 5	Day 7	Day 10
Creatinine (mg/dL)	8.47	7.06	3.41	1.94	0.78

**Table 2:** Relevant laboratory results.

	Erythrocyte sedimentation rate (ESR)	C- Reactive protein (CRP)	Antinuclear antibody	Acid fast bacterial culture (Tissue)
Result	28 mm/hr (0–20 mm/hr)	4.1 mg/dL (<1 mg/dL)	Negative	Negative

**Table 3:** Differential diagnosis of retroperitoneal fibrosis [4].

	Retroperitoneal fibrosis	Retroperitoneal lymphoma	Sclerosing mesenteritis	Desmoid-type fibromatosis	Inflammatory myofibroblastic tumor	Well-differentiated liposarcoma sclerosing variant
Ureteral obstruction	~80%	~50%	Rare	Rare	Rare	Unknown
Ureteral displacement	Medial	Lateral				
Aortic displacement	Rare	Anterior				
Reactive perivascular lymphoid aggregates	100%	Absent	Variable	Rare	Variable	Present in the inflammation type
Necrosis	Absent	Variable	Fat necrosis	Rare	Focal	Fat necrosis
Vasculitis	~50%	Absent	Absent	Absent	Absent	Absent
B-Catenin	Negative	Unknown	Negative	Positive in 90%	Negative	Variable
ALK-1	Negative	Negative	Negative	Negative	Positive in 50%	Negative
Desmin	Negative	Negative	Variable	Rare	Positive	Rare
S100	Negative	Negative	Negative	Rare	Negative	Positive in adipocytic component
